# HPTLC Fingerprinting—Rapid Method for the Differentiation of Honeys of Different Botanical Origin Based on the Composition of the Lipophilic Fractions

**DOI:** 10.3390/molecules23071811

**Published:** 2018-07-21

**Authors:** Ewa Makowicz, Izabela Jasicka-Misiak, Dariusz Teper, Paweł Kafarski

**Affiliations:** 1 Faculty of Chemistry, Opole University, Oleska 48, 45-052 Opole, Poland; makowicz.ewa@gmail.com; 2 Research Institute of Horticulture, Apiculture Division, ul. Kazimierska 2, 24-100 Puławy, Poland; dariusz.teper@inhort.pl; 3 Faculty of Chemistry, Wroclaw University of Science and Technology, ul. Wybrzeże Wyspiańskiego 27, 50-370 Wrocław, Poland; pawel.kafarski@pwr.edu.pl

**Keywords:** food quality, honey authentication, honey fingerprint, ultrasound assisted extraction, solid phase extraction, HPTLC

## Abstract

Bee honey possess various nutritional and medicinal functions, which are the result of its diverse chemical composition. The numerous bioactive compounds in honey come from flower nectar; thus, the identification of the specific chemical profiles of honey samples is of great importance. The lipophilic compounds from eight monofloral honeys (rape, buckwheat, clover, willow, milk thistle, dandelion, raspberry and sweet yellow clover) were investigated. Analyses of the lipophilic fractions obtained by UAE (ultrasound assisted extraction) and SPE (solid phase extraction) extractions were performed using high-performance thin layer chromatography (HPTLC). Chromatographic and cluster analyses allowed the identification of a unique, colorful pattern of separated compounds with specific Rf values on the HPTLC plate for each type of monofloral honey. HPTLC is a simple and effective visual method of analysis, and it can serve as a basis for authenticating different types of honey.

## 1. Introduction

Honey is a natural, sweet, aromatic and complex food product produced by honey bees (*Apis melifera* L.). Due to its taste and nutritional value, honey is commonly used in the food industry, in medicine and in the production of cosmetics [1,2,3]. The human use of honey can be traced to approximately 8000 years ago based on depictions in Stone Age paintings. Moreover, in every culture, evidence can be found that honey has been used as a source of food and as a symbolic item in religious, magic and therapeutic ceremonies [4].

The chemical composition of honey varies and is significantly dependent on multiple factors, such as the botanical and geographical origin, the bee species that produced the material, the age of the honey, the storage method and the honey processing procedures that were used (harvest technology and condition) [5,6,7]. These factors all influence the composition and properties of honey. In addition to the constant increasing consumer interest in natural and healthy food products, bee products have also grown in popularity. Therefore, controlling the quality, authenticity and nutritional value of such products has become extremely important [8]. In many countries (Germany, Australia, New Zealand, Italy, Croatia and Spain), control of honey quality is obligatory and is performed by accredited laboratories. Unfortunately, in most EU countries (including Poland), such controls are not obligatory. Even though the demand for honey in Poland and in other European markets is constantly increasing, cheaper honeys, which are characterised by poor quality, often make up a large fraction of those markets. Additionally, honeys are often falsified after harvesting by the addition of water, glucose–fructose syrups, corn syrups or by admixing with imported honeys of inferior quality [9,10,11].

Therefore, the evaluation of the quality and authenticity of honey is an important problem that needs to be solved by applied research, and the resolution will impact the food industry and consumer well-being. Information about the botanical and geographical origins of certain kinds of honey can be derived from the analysis of the content of its pollen grains, namely, by melissopalynology, which was proposed by French beekeeper J. Louveaux in 1978 [11]. This method relies on the microscopic analysis of bee pollen; however, the available literature is increasingly demonstrating that this method is not sufficient for the precise authentication of honey. However, honey could also be falsified by the intentional addition of the marker pollen [8,12].

There are essentially three approaches to basic studies aimed at the development of new, reliable methods for the identification of both the botanical and geographical origins of honeys as well as for establishing their quality. The approaches include (i) the search for individual compounds that are characteristic of certain honey brands, which could be considered their markers; (ii) the construction of profiles of chosen classes of compounds since, in numerous cases, they are characteristic of individual honeys and may serve as an individual honey fingerprint; and (iii) the application of metabolomics to study the whole metabolome of honeys and construct heat maps, which may serve as specific bar-codes characteristic of individual brands of honey [12,13,14,15,16]. The construction of honey profiles is mostly based on studies of the compositions of the volatile or phenolic fractions of honeys of different origins [14,17,18]. For this purpose, a wide variety of analytical procedures (LC-MS (liquid chromatography with mass spectrometry), HPLC (high performance liquid chromatography), gas chromatography with static/dynamic headspace analysis (HS/GC-MS), and solid-phase micro extraction (SPME) coupled to gas chromatography–mass spectrometry (GC–MS) have been used [18,19,20,21,22,23,24]. Spectroscopic methods such as NMR, FT-IR and NIR have also been successfully used for metabolomic studies [16,24,25,26]. There are limited reports about the usage of HPTLC [27,28], capillary electrophoresis [29], amperometric methods [30], ion chromatography [31] and even immunoassays [32]. Of course, the obtained results (including profiles and metabolomes) are strongly dependent on the isolation technique used to obtain the honey fraction and on the methods used to determine its composition.

The main purpose of this study was to use HPTLC for the differentiation of Polish honeys of different botanical origins. The fingerprint of each honey sample was created based on the lipophilic fraction of compounds extracted either by solid-phase extraction (SPE) or by ultrasound assisted extraction (UAE). These studies indicated the usefulness of the HPTLC method for the evaluation of different honey samples. Their specific fingerprints are mainly based on the similarities and differences in the observed Rf values, the colors of the spots and the profiles generated from the chromatograms. To the best of our knowledge, this work is one of the first reports on the use of HPTLC for the determination of the botanical origin of honey samples. So far, HPTLC procedures have been successfully applied in the differentiation of manuka and kanuka honeys [28] and for differentiation honeys based on the identified phenolic compounds [33]. Both of these works indicate the great potential of the usage of HPTLC for fast and accurate differentiation of honeys based on their botanical origin. Nonetheless, additional research is still required to enhance the potential of HPTLC usage for honey authentication.

## 2. Results and Discussion

To differentiate Polish honeys of different origin, two methods of extraction were applied, SPE and UAE. Each extract was analysed by HPTLC. Different sizes of plates (20 cm × 20 cm and 20 cm × 10 cm) and the mobile phase systems of varying compositions were initially tested (toluene-ethyl acetate (93:7); toluene-ethyl acetate (90:10); toluene-ethyl acetate (80:20) and toluene-ethyl acetate (70:30) (*v*/*v*). Additionally, two different derivatisation systems were tested (anisaldehyde and vanillin reagents). The best separation results for the differentiation of honeys of different botanical origin based on their lipophilic fractions were obtained when using toluene-ethyl acetate (80:20) as the eluent (*v*/*v*) and anisaldehyde as a derivatisation reagent. Additionally, different volumes of the analytes were tested (from 2 to 15 µL), and 10 µL was selected as the most appropriate volume. The HPTLC method was tested for the analysis of extracts from different monofloral Polish honeys, namely, buckwheat, raspberry, sweat yellow clover, clover, dandelion, milk thistle, willow and rape.

These studies were carried out with the understanding that the lipophilic fractions of honeys contain the varied classes of compounds, and thus the compounds might be useful for determining the botanical and geographical origins of honey as well as for establishing their quality. With increasing diversity of the analysed honeys and with different analytical approaches used throughout the world, it has become obvious that the identification of specific markers (certain compounds or sets of compounds characteristic for only one honey origin) has a low chance of success since most compounds are unspecific [17]. In the case of usage of HPTLC, it seems that we are able to determine the botanical origin of honeys, but not the adulterations. It could certainly act as a fast screening approach for testing honey samples.

**Figure 1:**
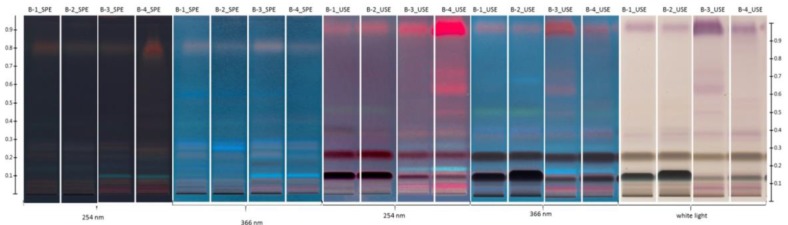
Represented HPTLC fingerprint of buckwheat honey after derivatisation (UAE—ultrasound solvent extraction, SPE—solid phase extraction visualised under white light, 254 nm and 366 nm; B1–B2 cv. Kora; B3–B4 cv. Panda; in Panda cv. we observed additional band on the chromatograms and, moreover, concentration of compound with Rf = 0.13 is less than in Kora cv.

**Figure 2:**
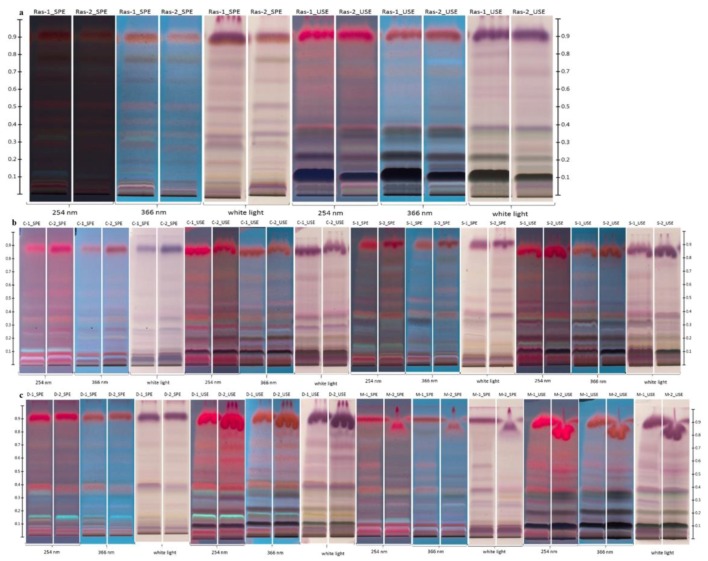
HPTLC fingerprints of honeys of different botanical origin after derivatisation (UAE—ultrasound assisted extraction, SPE—solid phase extraction visualised under white light, 254 nm and 366 nm; (**a**) Ras—raspberry honeys; (**b**) C—clover honeys; S—sweet yellow clover honeys; (**c**) D—dandelion honeys; M—milk thistle honeys).

**Figure 3:**
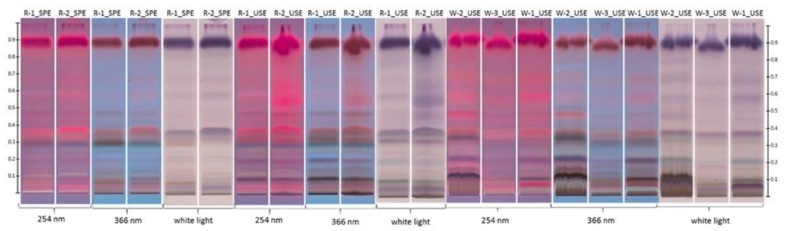
HPTLC fingerprint of rape and willow honeys after derivatisation (UAE—ultrasound assisted extraction, SPE—solid phase extraction visualised under white light, 254 nm and 366 nm; R—rape honeys; W—willow honeys.

In this study, we demonstrated that this HPTLC method could be a powerful tool for creating specific fingerprints of the composition of lipophilic fractions of honeys of different origins based only on visual differences in the plates. As is shown in Figure 1, when using the same extraction procedure, different patterns of bands were observed for different types of honeys. On the other hand, the patterns observed for specific types of honeys exhibit striking similarities (Figure 1, Figure 2 and Figure 3). Only the most important Rf values for differentiation of the tested honey samples are collected in Table 1.

All the performed analyses using both UAE and SPE methods indicated the utility of HPTLC for analysis of the lipophilic fractions of honey. Since the biggest differentiation between samples of different origins was observed in the HPTLC chromatograms obtained by derivatisation with anisaldehyde, we mainly focus on those results in this paper.

Figure 1, Figure 2 and Figure 3 show representative visual profiles of analysed extracts after SPE and UAE extraction. For clarity, only two samples from each honey type are shown, but it must be stated that the same fingerprints were observed for each test with a certain type of honey. It is worth noting that the majority of lipophilic fraction, especially volatile organic compounds which are important elements of studied extracts in both monofloral and polyfloral honeys are universal; therefore, for honeys of different botanical origin [5], the observed fingerprint, rather than the presence of the compounds, represents a unique pattern and is given by a set of Rf values and the specific colours observed for these compounds.

These results together allow the differentiation of honeys based on simple visual analysis. When the buckwheat honeys were analysed, some striking differences between the samples were observed. The differences are clearly visible from Figure 1 even though the botanical origin of all the samples was confirmed to be the same by pollen analysis. This result was obtained because two different varieties of buckwheat honey—*Fagopyrum esculentum* cv. Kora and *Fagopyrum esculentum* cv. Panda—exist in Poland and in other European countries. Upon microscopic analysis, their pollen grains look the same, and it is impossible to visually distinguish the varieties. However, the differences are clearly visible in the honey; honeys from *Fagopyrum esculentum* cv. Kora are darker than those from *Fagopyrum esculentum* cv. Panda, and the former is more widespread on the market.

In the case of raspberry honeys (Figure 2a), it seems that UAE extracts are much more indicative for the creation of this unique profile than the SPE one. Three dark and intensive spots (Rf = 0.33, Rf = 0.22 and Rf = 0.11) are characteristic. Nevertheless, for comparison with milk thistle honeys we can observe that only slight differences between them are observed in this case (e.g., presence of weak pink band with Rf = 0.28 in milk thistle honeys (UAE, 254 nm), which is not present in raspberry honeys). However, in this case, profiles after SPE extraction, visualised in different lights, could differentiate these two types of monofloral honey.

Moreover, by using HPTLC, we can easily identify differences between honeys in which the predominant pollens belong to plants from the same family. For example, clover (*Trifolium* spp.) and sweet yellow clover (*Mellilotus officinallis* L.) belong to the botanical family Fabaceae, and dandelion and milk thistle belong to the Astraceae family. The HPTLC fingerprints obtained for those honeys are present in Figure 2b,c. Honeys from the same botanical origin display almost the same fingerprints, which may complicate their proper identification. However, we noticed that some minor differences can be seen in their profiles. For example, in dandelion honeys, we observed a characteristic light blue band (after UAE and SPE in both 254 nm and 366 nm irradiation) with Rf = 0.16, and this band is not seen in milk thistle honeys. The slight differences observed among honeys of the same botanical origin are normal and are mostly the result of the presence of other kinds of pollen grains in the honeys. Moreover, other factors such as time of harvesting, method of processing or geographical origin may affect this unique pattern. Based on visual discrimination, it is also observed that the region with Rf values between 0.00 and 0.10, where good separation was not obtained for any of the testing methods, could also be a good indicator for honey discrimination. The colours of bands are usually different for honeys from different floral sources. It appears that in the future it will be possible to create rules for classification of monofloral honeys based on HPTLC patterns. It requires some time to analyse more samples to find the best method for isolation of compounds, which is a crucial step. Based on the discussed examples in this paper, this approach possesses great potential, which should be further developed. The HPTLC method could also be combined with a simple chemometric approach, such as HCA or PCA, to provide a rapid and useful method of identifying the botanical origin of honey samples.

**Figure 4:**
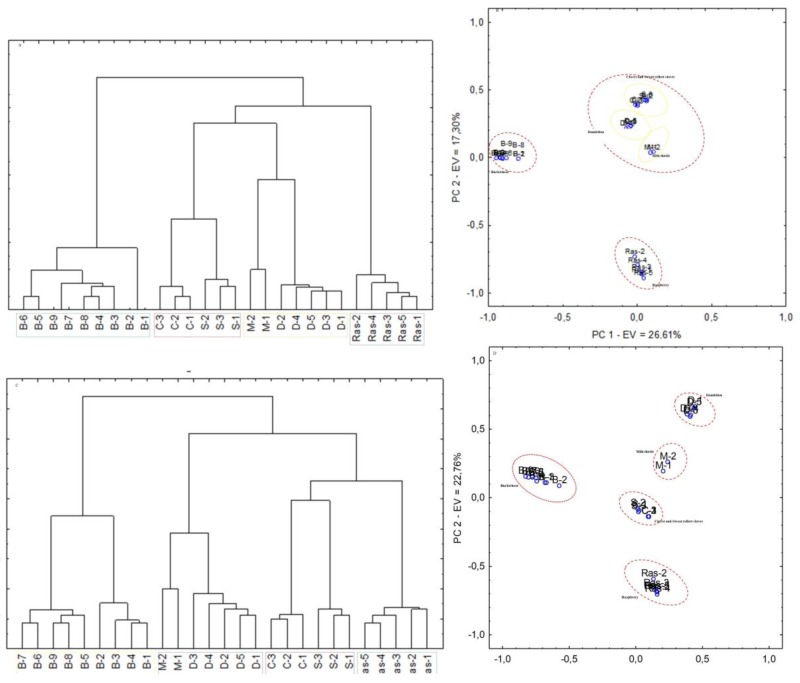
Exemplary statistical results for tested honey samples; (**a**) dendrogram after SPE under white light after derivatisation; (**b**) PCA score plot based on the results for SPE extracts under white light after derivatisation; (**c**) dendrogram after UAE method at 366 nm after derivatisation; (**d**) PCA score plot based on the results for UAE extracts at 366 nm after derivatisation; (Ras—raspberry honeys; D—dandelion honeys; M—milk thistle honeys; S—sweet yellow clover honeys; C—clover honeys; B—buckwheat honeys).

Exemplary results of HCA and PCA analysis for UAE and SPE extraction methods are presented in Figure 4. Analyses were performed to classify monofloral honey samples based on their composition of lipophilic compounds and HPTLC results. The results presented in Figure 4a–d clearly indicate that based on the obtained Rf values all honeys were clustered into separate groups according to their botanical origin. Moreover, both the SPE and UAE extracts of honeys in which the predominant pollens belong to plants of the same botanical family were clustered into single groups. However, best separation between groups was obtained for UAE extracts. Buckwheat and raspberry honeys cluster into separate groups. Additionally, only in HCA analysis for UAE extracts is observed separation between buckwheat honeys (Figure 4c). Based on the *Fagopyrum esculentum* cv. These results are comparable with the visual fingerprints of these honeys on the HPTLC plates.

Moreover, the presented results show that this HPTLC method could serve not only as a fast and very sensitive method for establishing the botanical origin of honeys but also as a method for detecting the adulterations caused by the beating of bee bread or by incorrect classification of the honey type. The pollen analysis of willow honey samples is a good example (Table 2).

According to the presented results, one of the willow honeys (W-1) is rape honey, while the second one (W-3) is probably adulterated by intentional application of willow pollen, which is reflected in its chemical composition (Figure 3).

HPTLC analysis revealed that W-3 has a different pattern than that of the other willow and rape honeys. These results show that the honey, which according to pollen analysis is classified as willow, is a polyfloral honey based on its chemical composition. In Figure 3, for extracts after UAE, a few characteristic bands for rape honeys were marked in yellow. The bands under white light with Rf = 0.11 (deep gran) and Rf = 0.21 (weak blue) were also present in the W-1 honey sample, which was incorrectly classified. Moreover, in W-2 samples, where *Salix* spp. was predominantly pollen, and *Brassica napus* L. pollen was the second one, with 20% abundance, the bands with Rf = 0.21 (weak blue) were also present. The insensitivity of bands was higher for those honeys where the rape pollen was seen in overwhelming amounts. Furthermore, it is interesting that the band with Rf = 0.34 (weak violet) is present only in rape honeys with a high percentage of *Brassica napus* L. (R-1 and R-2). This could be a discriminative factor for good classification of these two honey varieties. The presented results also indicate the usefulness of HPTLC method for differentiation of honeys, even if the percentages of the two types of pollens are relatively high.

**Figure 5:**
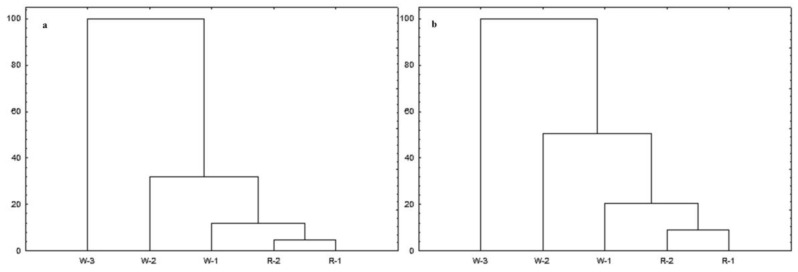
Dendrogram of cluster analysis of willow and rape honeys; (**a**) dendrogram after UAE in 366 nm; (**b**) dendrogram after SPE in 254 nm. (W—willow honeys; R—rape honeys).

Dendrograms (Figure 5a,b) obtained by hierarchical clustering analysis of willow and rape honeys show that the honeys were clustered in accordance with pollen analysis. Rape honeys, in which *Brassica napus* L. pollen was predominant and in relatively high amount, clustered into one group and were clustered alongside one of the honeys sold as a willow honey; the pollen analysis of that honey (W-1) indicated that it was another rape honey, but as we mentioned before, on HPTLC plates, some bands are missing compared to rape honeys with pollen amounts higher than 70%; therefore, after HCA it created a separate group. The sample of willow honey (W-2) that contained a high proportion of rape pollen (27%) (see Table 2) created a subgroup with the rape honeys. W-3 created a separate group that is not correlated with any other group. These results support the visual interpretation of the HPTLC results (Figure 3) where the observed pattern of bands for the W-1 and W-2 samples correlate with those obtained for the rape honeys.

## 3. Materials and Methods

### 3.1. Reagents and Materials

All chemicals used in this study were of analytical grade. Dichloromethane, methanol, toluene, ethanol, ethyl acetate, hydrochloric acid, 95% sulfuric acid, glacial acetic acid and anhydrous magnesium sulphate were purchased from POCH S.A. (Gliwice, Poland). *p*-Anisaldehyde (98%) was purchased from Sigma-Aldrich (Poznań, Poland). Silica gel 60 HPTLC aluminum plates (20 cm × 20 cm) were purchased from Merck (Kennborough, NJ, USA), and Strata-SDBL SPE cartridges were purchased from Phenomenex (Torrance, CA, USA).

### 3.2. Honey Samples and Melissopalynological Analyses

A total of 32 honey samples representing 8 different botanical origins, namely, rape (*Brassica napus* L.), buckwheat (*Fagopyrum esculentum* Moench), clover (*Trifolium* spp.), willow (*Salix* spp.), milk thistle (*Silybum marianum* (L.) Gaertner), dandelion (*Taraxacum* spp.), raspberry (*Rubus* spp.) and sweet yellow clover (*Melilotus officinalis* L.), were analysed in this study. The analysed honey samples were purchased between 2013 and 2016 from small, domestic apiaries located in different locations in Poland; the apiaries reported the botanical origin of the honeys. Before analysis, each honey sample was subjected to pollen analysis. Analyses were performed on an Olympus BX41 microscope (Olympus America, PA, USA) under 400× magnification according to the methodology recommended by the International Commission of Bee Botany and by the International Honey Commission (Louveaux et al. 1978). Honeys were stored at 4 °C in the dark before analysis.

### 3.3. Extraction of Lipophilic Fraction from Honeys

For HPTLC separation of the lipophilic fraction, two methods of extraction were used, UAE and SPE. The biggest advantage of these methods is that they do not require heat, so the problems with the appearance of artefacts, which are mainly caused by Maillard reactions and oxidations, are avoided. Both methods are more efficient than liquid–liquid extraction, and they are the most appropriate for the isolation of the lipophilic compounds of honeys, which comprise an important group of compounds in the obtained extract. Furthermore, it is well known that honeys of different botanical origins exhibit very similar compositions to these compounds, which is crucial during the development of the method, which is based on the chemical composition of the obtained fractions. Additionally, both methods used were optimised in our previous research.

#### 3.3.1. Ultrasound Assisted Extraction (UAE)

UAE was performed as described previously [13] with some modifications. Forty grams of honey sample was dissolved in 22 mL of distilled water and placed in a 150-mL flask, and 1.5 g of anhydrous magnesium sulfate was then added. Subsequently, 20 mL of dichloromethane was used as an extraction solvent. UAE was performed in an ultrasonic cleaning bath (Cole-Parmer 8891) at 25 °C for 30 min. At the end of each sonication period, the whole extract was centrifuged at 3000 rpm, and the organic layer was collected. Each sample was extracted three times. The organic layers were combined, dried over anhydrous magnesium sulfate, and concentrated on a rotary evaporator at 35 °C. The entire procedure was performed three times for each sample with the same amount of honey. The resulting oily residues were dissolved in 500 µL of dichloromethane and stored at 4 °C until HPTLC analysis.

#### 3.3.2. Solid Phase Extraction (SPE)

SPE was performed as described previously [13] with some modifications, and it was carried out in a Baker SPE-12G vacuum manifold (J. T. Baker^®^, Phillipsburg, NJ, USA) at a flow rate of 2 mL/min. For extraction, Strata–SDBL cartridges were used with 200 mg of styrene-divinylbenzene resin (Phenomenex). Prior to use, the cartridges were conditioned by rinsing them with 4 mL of dichloromethane, 4 mL of water, and 4 mL of an ethanol–water mixture (12%, *v*/*v*). Then, 15 g of honey was dissolved in 25 mL of water and passed through the cartridges. After this, the remaining sugars and other hydrophilic components were flushed out with 20 mL of water. Finally, elution was performed with 20 mL of dichloromethane. The obtained organic extracts were dried over anhydrous magnesium sulfate and concentrated on a rotary evaporator at 35 °C. Residues were dissolved in 350 µL of dichloromethane and stored at 4 °C until HPTLC analyses. The entire procedure was performed three times with the same amount of each sample.

### 3.4. HPTLC Separation of Honey Extracts

HPTLC analyses of all the obtained extracts of the lipophylic fraction of the honeys were performed on precoated silica gel 60 HPTLC aluminium plates (20 cm × 10 cm) purchased from Merck. Ten microliters of each honey extract (with concentration 100 ± 5 ng/spot) was applied to the plate in 10 mm bands 15 mm from the lower edge of the plate at a rate of 250 nL·s^−1^ using a semi-automated HPTLC application device (Linomat 5, CAMAG, Muttenz, Switzerland) under a flow of N_2_ gas. The chromatographic separations were performed in a chromatographic tank saturated for 20 min with the mobile phase (toluene-ethyl acetate, 80:20 (*v*/*v*)) and developed to a distance of 90 mm. The obtained results were documented using an HPTLC imaging device (TLC Visualizer, CAMAG, Muttenz, Switzerland) under white light and at 366 nm and 254 nm.

#### Derivatisation

Following the chromatographic separation and documentation, the plates were derivatised with an anisaldehyde reagent (prepared by adding 10 mL of glacial acetic acid to 0.5 mL of anisaldehyde and dissolving the mixture in 85 mL of methanol, and then adding 10 mL of concentrated sulfuric acid dropwise to the solution). After derivatisation, the HPTLC plates were dried at 105 °C for 5 min, and the resulting bands were observed under white light and at 366 nm. The obtained chromatographic images were analysed by using HPTLC software (visionCATS, CAMAG).

### 3.5. Statistical Analysis

For obtained images of the HPTLC plates Rf values for bands were calculated with winCATs software. The obtained data were used in hierarchical cluster analysis (complete linkage using Euclidean distances) and for principal component analysis (PCA). Both of these classification techniques were used to examine the trend in the data and to discover the natural grouping of the tested honey samples. Statistical analyses were performed using STATISTICA 13.1 software (StatSoft Inc., Tulusa, OK, USA).

## 4. Conclusions

The obtained results show that HPTLC is a very sensitive visual method for the rapid and precise differentiation of honeys of different botanical origins, since they generate bar-code-like images. This is because the extracts of the lipophylic fractions obtained by UAE and SPE create a specific pattern of separated compounds for each type of monofloral honey. This pattern is generated according to highly specific Rf values and the coloration of the compounds. These results, along with cluster analysis, seem to be specific enough to provide unique and characteristic fingerprints of honeys of different botanical origins. It seems that this method is sensitive and precise enough to compete with GC-MS techniques, even when the structures of the chemical constituents present in the specific extracts are not determined. Additionally, as indicated by isolated examples, this method could be effectively used for the determination of some of the possible falsifications of monofloral honeys—e.g., intentionally addition of pollen after harvesting honey, which would change the origin. Based on the statistical analysis, it seems that UAE extraction method is more adequate for lipophilic fraction profile creation, since the differences between samples are more significant and better separation is achieved.

## Figures and Tables

**Table 1:** Characteristic Rf values for tested honeys.

Honey	Derivatised UV		366 nm Derivatised		254 nm Derivatised		Derivatised UV		366 nm Derivatised		254 nm Derivatised	
UAE							SPE					
	*R_f_	Zone Color	*R_f_	Zone Color	*R_f_	Zone Color	*R_f_	Zone Color	*R_f_	Zone Color	*R_f_	Zone Color
Buckwheat (cv.KORA) (*Fagopyrum esculentum* Moench)	0.35 0.23 0.11	weak violet brown deep green	0.45 0.23 0.11	weak green brown deep brown	0.23 0.34 0.45 0.11	brown orange weak green deep brown	0.05 0.28 0.08	weak orange pink weak brown	0.60 0.42 0.33 0.09	blue weak gray blue orange	0.08 0.18 0.33 0.43	orange deep orange weak brown weak green
Buckwheat (cv.PANDA) (*Fagopyrum esculentum* Moench)	0.35 0.23 0.11 0.05	weak violet brown deep green pink	0.45 0.23 0.11 0.14 0.05	weak green brown deep brown light blue pink	0.14 0.05 0.11 0.23 0.45	weak blue pink deep brown brown weak green	0.08 0.04 0.19 0.28	weak brown violet weak brown pink	0.42 0.33 0.12 0.18 0.08 0.05	weak gray blue blue brown orange pink	0.08 0.18 0.12 0.33 0.43	orange deep orange blue weak brown weak green
Raspberry (*Rubus* spp.)	0.11 0.19 0.22 0.33	deep black weak brown gray green	0.11 0.13 0.19 0.33	deep black deep pink deep brown deep violet	0.11 0.13 0.19 0.23 0.70	deep black pink deep brown weak blue grey	0.13 0.34 0.75	orange violet weak brown	0.20 0.28 0.34 0.67 0.75	violet weak violet weak brown weak orange brown	0.13 0.67 0.75	weak blue gray brown
Sweet yellow clover (*Melilotus officinalis*)	0.08 0.11 0.17 0.22 0.29 0.39	deep violet deep brown yellow weak brown weak violet violet	0.08 0.11 0.22 0.31 0.39 0.36	deep violet deep purple weak brown pink orange weak green	0.08 0.11 0.22 0.31 0.39 0.36	deep pink deep purple brown pink orange blue	0.05 0.15 0.37	violet yellow violet	0.05 0.14 0.32	deep pink weak blue gray	0.08 0.12 0.15 0.37 0.46	weak pink weak brown weak gray orange pink
Clover (*Trifolium* L.)	0.08 0.11 0.22 0.29 0.39	deep violet deep brown weak brown weak violet violet	0.08 0.11 0.22 0.31 0.39	deep violet deep purple weak brown pink orange	0.08 0.11 0.22 0.31 0.39	deep pink deep purple brown pink orange	0.06 0.11 0.30	deep violet gray violet	0.06 0.11 0.23 0.36	weak blue pink weak orange weak orange	0.06 0.11 0.13 0.26 0.29	pink deep pink weak blue weak pink pink
Dandelion honey (*Taraxacum* spp.)	0.15 0.40 0.36	yellow deep yellow violet	0.10 0.22 0.33 0.38 0.48	deep purple brown brown deep pink weak gray	0.10 0.16 0.21 0.38	deep purple blue brown deep pink	0.10 0.17 0.22 0.37	green deep yellow weak green deep pink	0.15 0.31 0.37 0.40	blue weak brown deep pink gray	0.10 0.16 0.37	gray blue deep pink
Milk thistle (*Silybum marianum* (L.) Gaertner)	0.11 0.22 0.29 0.36 0.39	deep green weak brown weak violet orange violet	0.11 0.22 0.36 0.49	deep purple weak brown orange weak brown	0.11 0.28 0.22 0.49 0.36	deep purple weak pink brown gray orange	0.13 0.28 0.32 0.36	weak gray weak violet violet orange	0.11 0.25 0.28 0.36 0.48	deep pink weak gray gray orange weak brown	0.07 0.14 0.25 0.36	deep pink weak blue weak gray orange
Rape (*Brassica napus* L.)	0.11 0.21 0.34	deep green weak blue weak violet	0.11 0.21 0.31 0.39 0.49	deep brown gray deep gray pink gray	0.11 0.22 0.31 0.40 0.50	brown weak brown gray pink gray	0.36 0.49	weak green weak orange	0.09 0.39 0.49	weak orange pink gray	0.09 0.21 0.31 0.49	weak orange weak pink green gray

UAE—ultrasound solvent extraction; SPE—solid phase extraction. *Rf values are average from three plates for each extract and SD (=standard deviation) for each value was less than 0.02.

**Table 2:** Characterisation of the analysed rape and willow honeys.

Samples	Declared Botanical Origin	Predominant Pollen (%) ^a^		Botanical Origin
R-1	Rape	89 6	*Brassica napus* L. *Salix* spp.	Rape honey
R-2	Rape	73 18	*Brassica napus* L. *Salix* spp.	Rape honey
W-1	Willow	61 27	*Brassica napus* L. *Salix* spp.	Rape honey
W-2	Willow	59 20	*Salix* spp. *Brassica napus* L.	Willow honey
W-3	Willow	65 10	*Salix* spp. *Rubus* spp.	Willow honey *

^a^ average calculated from two independent analyses. * According to melissopalynological analysis of the honey, nectar composition might be different because of possible adulteration by beating of bee bread.
